# Coronilla Varia
*Poulet, Socièté de Therapeutique, *Les Nouv. Rem.* Oct. 24th; 1891 Bull, *Gen de Ther.*, Dec. 15th, 1891; *Therap. Gaz.*, Mar. 15th 1892.


**Published:** 1892-08-06

**Authors:** 


### THERAPEUTICS.

### CORONILLA VARIA.*

Coronilla is a cardiac remedy, especially useful in paroxysmal tachycardia, and its action has been investigated by Dr. V. Poulet, of Plancher-les-Mines. It has only rarely been utilised for its therapeutical properties, and in some works it has been stated that it is poisonous. Kirschleger, in his "Flora of Alsace," says that it is a reputed purgative and diuretic. These properties are assigned to it on the vaguest grounds, for its reputation in these respects is not founded on any clearly proved facts. Dr. Poulet calls attention to the fact that in 1888 Schlagdenhauffen and Leeb studied this substance chemically and physiologically, and

* Poulet, Société de Therapeutique, *Les Nouv. Rem.* Oct. 24th; 1891 Bull, *Gen de Ther.*, Dec. 15th, 1891; *Therap. Gaz.*, Mar. 15th 1892.

isolated from it an active principle called coronilline, which is a glucoside. The tincture has been employed with excellent results as a powerful cardiac tonic, causing increased arterial tension, strengthening the pulse, increasing diuresis, decreasing œdema and dyspnœa. Its place would seem to be in the treatment of cases where digitalis is badly borne, and where digitalis causes gastric or intestinal irritation, or when it has lost its efficacy. Sometimes, however, coronilla scorpioides itself sets up vomiting or diarrhœa.

The investigator states that he has employed the tincture of coronilla with most excellent results in the treatment of paroxysmal tachycardia, being able to overcome the extremely frequent pulse, and in some cases the more or less violent precordial pain, and other symptoms which are associated with this affection, particularly in those cases which depend upon disease in the aortic or mitral valves. He reports a case of paroxysmal tachycardia, combined with mitral regurgitation, in a lady aged 49, showing the efficacy of coronilla to prevent the return of the attacks.

The remedy is very beneficial in cases of cardiac asthma; in such cases the appetite improves, and the gastro-intestinal tract is stimulated. In the case of acute hyperæmia of the lung, with tachycardia, agonising dyspnœa, and danger of fatal asphyxia, he employed coronilla with the result of obtaining rapid resolution and the avoidance of all complications. The dose under these circumstances was two and a half drachms of the tincture in eight hours, the patient being a man of 64 years, who had had frequent attacks of broncho-pneumonia and rheumatism. When first seen there was very marked dyspnœa, high fever, severe cough, and expectoration of a large quantity of glairy mucus; the respirations were forty-four per minute, and the pulse varied in frequency, but reached as high as 180 per minute, being small and exceedingly difficult to count. The tongue was coated and nausea was present. On auscultation the respiratory murmur was very feeble, and there was diminished resonance on the right side. He was immediately ordered the tincture of coronilla, given in divided doses every hour. On the following day the patient was wonderfully improved, and passed a good night. His condition was excellent, and he presented a marked contrast to the anxious state of the day before. The tongue was clean, the cough decreased, and the expression natural. The respiration was twenty per minute and the pulse eighty, strong and regular, while the respiratory and cardiac sounds were normal; to use the words of the reporter, it was a complete metamorphosis.

Another case recorded by M. Poulet illustrates the action of coronilla in paroxysmal tachycardia with violent pain. C. G., age fourteen, belonged to a family of cardiac patients. His mother was affected with diseased heart, and a maternal aunt, as well as other relatives. He himself suffered from palpitation; he could not rise without being very much embarassed. There was no bruit audible on auscultation, but the impulse was very forcible, and the precordial area of dulness greatly enlarged. For eight days he experienced nocturnal attacks, accompanied by tachycardia and violent pain in the precordial area and epigastrium, which came upon him repeatedly, sometimes causing complete insomnia. He was ordered sixty drops of the tincture of coronilla varia, three times daily. From the first day the painful attacks of tachycardia ceased. The remedy was continued for eight days, without any new feature being observed. But on the ninth day the little fellow, having discontinued the remedy of his own accord, was subject to the attacks during the night, which again left him on the morrow when the treatment was resumed.

In a case of sunstroke, complicated by gastric and circulatory disturbance, results just as favourable were obtained. The patient was a man aged 35, who suffered greatly from frontal headache and bilious vomiting. The vertigo was continual, and the condition of the patient grave. The pulse was 90, and irregular, and the heart's action arbythmical. The tincture of coronilla was given in forty-five minimum doses daily for one week. This was followed by very marked improvement; the vertigo disappeared, the appetite improved, and the patient considered he was convalescent.

Dr. Poulet regarded coronilla as especially servicable in patients worn out and saturated with digitalis, which often has a deleterious action in the gastro-intestinal mucous membrane.

The dose of coronilline is from twenty to sixty centigrammes, and of the extract of coronilla, a half to one gramme and a half. These remedies should be given in pilules or cachets, for they are most unpleasant in flavour.

